# Commentary: CVM-1118 (foslinanib), a 2-phenyl-4-quinolone derivative, promotes apoptosis and inhibits vasculogenic mimicry via targeting TRAP1

**DOI:** 10.3389/pore.2026.1612325

**Published:** 2026-01-27

**Authors:** Mary J. C. Hendrix, Elisabeth A. Seftor, Richard E. B. Seftor, Yi-Wen Chu, Du-Shieng Chien, Yen-Ling Chen

**Affiliations:** 1 Shepherd University, Shepherdstown, WV, United States; 2 TaiRx, Inc., Taipei, Taiwan

**Keywords:** CVM-1118, CVM-1125, HIF1α, TRAP1, vasculogenic mimicry

## Introduction

In this issue of Pathology & Oncology Research [1], Shen and colleagues report on a novel drug, CVM-1118 (generic name foslinanib), which is a phosphoric ester compound selected from 2-phenyl-4-quinolone derivatives. The major active metabolite of CVM-1118 is CVM-1125, which has shown inhibitory and cytotoxic effects at nanomolar range. Additional new insights regarding the molecular effects of CVM-1118 have revealed its ability to inhibit tumor cell growth, induce apoptosis and cell cycle arrest, as well as inhibit vasculogenic mimicry – which describes the ability of aggressive tumor cells to form *de novo* vascular perfusion networks. In depth molecular screening and biological analysis indicate that CVM-1118 targets the TNF receptor associated protein 1 --TRAP1, via its metabolite CVM-1125.

**FIGURE 1:**
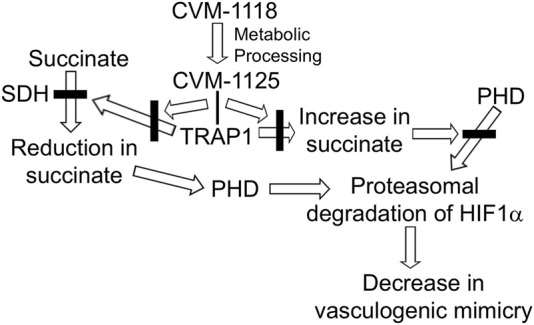
TRAP1 stabilizes HIF1a by inhibiting succinate dehydrogenase (SDH), which results in the accumulation of succinate. The increase in succinate inhibits prolyl hydrolases (PHD) from promoting the proteasomal degradation of HIF1a by modifying hydroxyl groups on HIF1a. CVM-1118, via its metabolite CVM-1125, binds TRAP1 and prevents it from inhibiting SDH. The resulting decrease in succinate levels promotes the degradation of HIF1a and subsequent reduction in vasculogenic mimicry.

The authors further examined the complex signaling pathways underlying tumor cell growth, plasticity, and metastasis, by employing an armamentarium of *in vitro* and *in vivo* analyses. The results collectively led to the discovery that TRAP1 stabilizes HIF1α by inhibiting succinate dehydrogenase (SDH), which results in the accumulation of succinate. The increase in succinate inhibits prolyl hydrolases (PHD) from promoting the proteasomal degradation of HIF1α by modifying hydroxyl groups on HIF1α. Most noteworthy, the molecular mechanisms underlying CVM-1118’s effects involve the direct binding of CVM-1125 to TRAP1, preventing it from inhibiting SDH -- resulting in a reduction in succinate, which leads to the degradation of HIF1α and subsequent reduction in vasculogenic mimicry. The major steps describing these molecular mechanisms of action are summarized in Figure 1. These experimental findings provide the first evidence demonstrating CVM-1118 as a novel anti-cancer drug worthy of further consideration and advancement through clinical trials – and identify the key signaling pathways responsible for the pharmacological effects.

## Discussion

Targeting tumor growth and metastasis has been a challenging endeavor for cancer researchers and oncologists. Over time, several approaches have been executed – each with their own merit, that have incrementally advanced the field. These include, but are not limited to: radiation therapy, hormone therapy, chemotherapy, angiogenesis inhibitor therapy, targeted molecular therapies, and immunotherapy. Although many patients have benefitted from these types of therapies, the most aggressive forms of cancer evolve as resistant over time, suggesting that plasticity and heterogeneity must be considered as a major factor in developing the most effective customized approach.

A noteworthy example of tumor cell plasticity is vasculogenic mimicry, originally described in human metastatic melanoma [2], and then validated across a wide spectrum of aggressive forms of cancer [3]. At the time this new concept was introduced, angiogenesis inhibitor therapy – targeting newly formed angiogenic vasculature, was considered novel [4]. However, many patients with aggressive forms of cancer did not fully respond to this new therapy [5, 6]. We hypothesized that tumor cells engaged in vasculogenic mimicry, which allows the rapid perfusion of a tumor through the creation of vascular networks, do not express the same molecular profile as endothelial cells forming angiogenic vessels – although they exhibit a similar perfusion function. We validated this hypothesis by treating aggressive tumor cells with various angiogenesis inhibitors and observed that vasculogenic mimicry was not significantly inhibited, due to the absence of the receptor(s) for the angiogenic therapy [7]. This is particularly noteworthy because the tumor cell formed perfusion pathway also provides a route for metastatic dissemination. Therefore, it was important to search for a drug or combination of agents that could effectively neutralize tumor cell vasculogenic mimicry.

CVM-1118 is a potential first-in-class drug for inhibiting tumor growth and metastasis via inhibition of vasculogenic mimicry. This drug is currently in Phase lla clinical trials – and shows promising signs as a novel anti-cancer drug in advanced hepatocellular carcinoma and neuroendocrine tumors. In addition, CVM-1118 (foslinanib) has attained orphan drug status for patients with pancreatic neuroendocrine tumors [8]. Moreover, combinatorial strategies including CVM-1118 are being pursued to target the heterogeneous components of a variety of aggressive tumors engaged in vasculogenic mimicry. It is exciting to consider that we are realizing the potential of translational research – from basic discovery to clinical application. This is due to the collective efforts of pathologists, basic researchers, and clinician scientists – providing new approaches for effectively targeting the most aggressive forms of cancer.

## Data Availability

Publicly available datasets were analyzed in this study. This data can be found here: Shen L, Chen YL, Huang CC, Shyu YC, Seftor REB, Seftor EA, et al. CVM-1118 (foslinanib), a 2-phenyl-4-quinolone derivative, promotes apoptosis and inhibits vasculogenic mimicry via targeting TRAP1. Pathol Oncol Res (2023) 29:1611038. doi:10.3389/pore.2023.1611038.
